# Dataset on the conceptualization of institutional trust on intention to participate in family takaful products

**DOI:** 10.1016/j.dib.2023.109808

**Published:** 2023-11-14

**Authors:** Mohd Faizuddin Muhammad Zuki, Nashirah Abu Bakar

**Affiliations:** a Islamic Business School, College of Business, Universiti Utara Malaysia, 06010 Sintok, Malaysia; b Faculty of Muamalat & Islamic Finance, Kolej Universiti Islam Perlis, 02000 Kuala Perlis, Malaysia

**Keywords:** Family Takaful (Solidarity) products, Institutional trust, Behavioural intention, Participation, Islamic insurance

## Abstract

The dataset was obtained from a time cross-sectional empirical survey to determine the intention to participate in Family Takaful (Solidarity) products. The survey was collected from 277 academicians in private Islamic Higher Education Institutions in Malaysia, with 272 valid cases. The academicians were chosen because of the various education levels, as education is an influential factor in many previous behavioural studies. The questionnaire was developed using scales based on the previous literature, such as intention, attitude, subjective norms, perceived behavioural intention and institutional trust. The reliability and validity of all variables indicated that the measurement items were appropriate. This dataset brought a novelty by conceptualizing the institutional trust construct to predict the customers’ intention to participate in Family Takaful (Solidarity) products. Results indicated that the data is suitable for performing replication studies.

Specifications Table

**Table:** 

Subject	Behavioural studies & Islamic finance
Specific subject area	Behavioural studies on intention to participate in Family Takaful (Solidarity) products. Specifically, the dataset emphasized customers’ intentions, attitudes, subjective norms, perceived behavioural control and institutional trust.
Data format	Raw, Analyzed, Filtered
Type of data	Table, Figure
Data collection	The data was collected from a time series of cross-sectional empirical surveys to examine the customer's intention to participate in Family Takaful (Solidarity) products. The survey was collected from 277 academicians in private Islamic Higher Education Institutions in Malaysia, with 272 valid cases. The reliability and validity of all variables indicated that the measurement items were appropriate. This dataset brought a novelty by conceptualizing the institutional trust construct to predict the customers’ intention to participate in Family Takaful (Solidarity) products. Results indicated that the data is suitable for performing replication studies.
Data source location	City/Town/Region: Private Islamic Higher Education Institutions Country: Malaysia
Data accessibility	Mendeley DOI: 10.17632/tp8rz425b9.1 Direct URL to data: https://data.mendeley.com/datasets/tp8rz425b9/1

## 1 Value of the Data

• Takaful operators should benefit from this dataset as it illustrates the important role of institutional trust in influencing the customer's intention to participate in Family Takaful (Solidarity) products. This finding suggests that customers’ trust toward the institution (Takaful operators), such as guarantees, contracts, regulations, promises, legal re-course, processes, or procedures of a Takaful company, is in place to protect them.
• The dataset is also appropriate for replicating the study in other regions or with other types of potential customers. The replication of the study will verify the goodness of fit of the scale used in this study.
• The adaptation of the institutional trust construct is also specifically conceptualized in the scope of Islamic financial services (Family Takaful (Solidarity) products). Thus, the measurement items could be used for behavioural studies in other Islamic financial services such as Islamic mortgages, Islamic banking products, Islamic investment instruments, etc.

## 2 Background

The dataset has been collected through a time series of cross-sectional empirical surveys to examine the customer's intention to participate in Family Takaful (Solidarity) products. This study brought its uniqueness by conceptualizing the institutional trust construct as a mediator of relationships between endogenous and exogenous variables. To the best of the researcher's knowledge, few previous empirical works have examined the role of institutional trust in accepting or participating in Islamic financial products and services, specifically Family Takaful (Solidarity) products. Thus, the empirical results from this study provided a better understanding of the role of institutional trust in influencing the customer's intention to participate in the products.

## 3 Data Description

The dataset was collected from a time series of cross-sectional empirical surveys to examine the customer's intention to participate in Family Takaful (Solidarity) products. The three constructs, attitude, subjective norms, and perceived behavioural control were adopted from the Theory of Planned Behaviour (TPB) [1]. This study conceptualizes a new construct as a research novelty: institutional trust. This construct is based on the Initial Trust Formation Model (IFTM) [2]. The institutional trust construct has been conceptualized as a mediator of the study in influencing the relationships between endogenous and exogenous variables. Table 1 presents the operational definitions for each of the study's constructs.

**Table 1:** Operational definitions of the constructs.

No.	Variable	Definition
1.	Intention to Participate in FTP	An individual's subjective probability of participating in Family Takaful (Solidarity) products [1,3].
2.	Attitude	An individual's positive or negative evaluation to participate in Family Takaful (Solidarity) products [1].
3.	Subjective Norms	An individual's perception of the social pressures placed on them to participate or not to participate in Family Takaful (Solidarity) products [1].
4.	Perceived Behavioural Control	An individual's perception of the ease or difficulty to participate in Family Takaful (Solidarity) products [4].
5.	Institutional Trust	An individual's belief of structural assurances such as guarantees, contracts, regulations, promises, legal re-course, processes or procedures of a takaful company is in place to protect them [2,^5^,6]

The measurement item of questionnaires is presented in the following tables, which consist of two parts. The first part consists of categorical items on the demographic profile of respondents, while the second part consists of ten – Likert scale items on endogenous, exogenous and mediator variables. The measurement items on the demographic profile (Q1 – Q7) are presented in Table 2, and the multi-scale items (Q8 – Q12) measurement is in Table 3. All multi-scale items are adopted from past literature works. For instance, measurement items for attitude adopted from [7], [8], [9], subjective norm [9,10], perceived behavioural control [7,^9^,^11^,12], institutional trust [5,13] and intention [9,^10^,14].

**Table 2:** Items for the demographic profile of respondents.

Variable	Questions	Coding
**Q1**	**Gender**	
	Male	1
	Female	2

**Q2**	**Marital Status**	
	Married	1
	Single	2

**Q3**	**Age**	
	21 – 30 years	1
	31 – 40 years	2
	41 – 50 years	3
	> 50 years	4

**Q4**	**Highest education level**	
	Bachelor's degree	1
	Master's degree	2
	PhD	3

**Q5**	**Monthly income**	
	RM2501 – RM4000	1
	RM4001 – RM5500	2
	> RM5501	3

**Q6**	**Year of services**	
	Less than 5 years	1
	6 – 10 years	2
	11 – 15 years	3
	16 – 20 years	4
	> 21 years	5

**Q7**	**Name of Institutions**	
	Universiti Islam Selangor (UIS)	1
	Albukhary International University (AIU)	2
	International Centre for Education in Islamic Finance (INCEIF)	3
	Universiti Islam Antarabangsa Sultan Abdul Halim Mu`adzam Shah (UniSHAMS)	4
	Universiti Sultan Azlan Shah (USAS)	5
	Universiti Islam Pahang Sultan Ahmad Shah (UniPSAS)	6
	Universiti Melaka (UNIMEL)	7

**Table 3:** Items for multi-scale items.

Vari- able	Questions	Coding
**Q8**	**Attitude (*Bhatti & Md Husin, 2019; Shih & Fang, 2004; Taylor & Todd, 1995*)**	
ATT1 ATT2 ATT3 ATT4 ATT5 ATT6 ATT7 ATT8	For me, to participate in family takaful products is a good decision. For me, family takaful product enables me to solve my financial problems. For me, saving packages in family takaful products will be beneficial in the future. I think family takaful product is a valuable package. I think family takaful product is a good protection package. I prefer a family takaful product that provides a saving plan. I prefer a family takaful product that provides an investment plan. I would be happy to protect myself with family takaful products.	(1 – 10) Likert (1 – 10) Likert (1 – 10) Likert (1 – 10) Likert (1 – 10) Likert (1 – 10) Likert (1 – 10) Likert (1 – 10) Likert

**Q9** SN1 SN2 SN3 SN4 SN5 SN6 SN7 SN8	**Subjective Norm (*Md Husin et al., 2016; Taylor & Todd, 1995*)** My family members, who influenced my decision, think I should participate in a family takaful product. My family members, who are important to me, think I should participate in a family takaful product. My family, who influenced my behaviour, thinks I should participate in a family takaful product. My office mates suggested that I should participate in a family takaful product. My best friends think that I should participate in a family takaful product. The takaful agent thinks that I should participate in a family takaful product. The takaful agent thinks I should participate in a family takaful product to protect myself and my family from unforeseen risks. The takaful agent influenced my decision to participate in a family takaful product.	(1 – 10) Likert (1 – 10) Likert (1 – 10) Likert (1 – 10) Likert (1 – 10) Likert (1 – 10) Likert (1 – 10) Likert (1 – 10) Likert

**Q10** PBC1 PBC2 PBC3 PBC4 PBC5 PBC6 PBC7 PBC8	**Perceived Behavioural Control (*Bhatti & Md Husin, 2019; Compeau & Higgins, 2017; Tan & Teo, 2000; Taylor & Todd, 1995*)** It will be easy for me to participate in a family takaful product soon. I am very confident that I can participate in a family takaful product soon. I have total control over participating in a family takaful product. If I wanted to, I could easily participate in any family takaful products on my own. I could participate in a family takaful product even if there was no one around to show me how to do it. I am confident to participate in a family takaful product if only by referring to the takaful's website. I am confident of participating in a family takaful product even if I have never participated in such a product before. I am confident of participating in a family takaful product if I have just seen someone participate in it before participating in it myself.	(1 – 10) Likert (1 – 10) Likert (1 – 10) Likert (1 – 10) Likert (1 – 10) Likert (1 – 10) Likert (1 – 10) Likert (1 – 10) Likert

**Q11** IT1 IT2 IT3 IT4 IT5 IT6 IT7 IT8	**Institutional Trust (*Ennew et al., 2011; Moin et al., 2015*)** My confidence and trust in the Takaful companies are very high. I believe that family takaful companies act honestly and ethically. I am confident that the policies and rules of a family takaful company protect its customers. Takaful companies that offer family takaful products are very reliable. The family takaful company has a good reputation. I believe that family takaful companies comply with Islamic financial laws. I believe that family takaful companies are going to make sure their agents are trained and professional. I believe that the administrative system of a family takaful company is efficient and effective.	(1 – 10) Likert (1 – 10) Likert (1 – 10) Likert (1 – 10) Likert (1 – 10) Likert (1 – 10) Likert (1 – 10) Likert (1 – 10) Likert

**Q12** INT1 INT2 INT3 INT4 INT5 INT6 INT7 INT8	**Intention (*Madden et al., 1992; Md Husin et al., 2016; Taylor & Todd, 1995*)** I plan to participate in a family takaful product in future. I intend to join a family takaful product if I have strong financial ability. I plan to protect my family with a family takaful product. I expect to participate in a family takaful product. I want to participate in a family takaful product. If given the opportunity, I will participate a family takaful product in the future. I will make some effort to participate in a family takaful product in future. I will strongly recommend a family takaful product for others to participate in it.	(1 – 10) Likert (1 – 10) Likert (1 – 10) Likert (1 – 10) Likert (1 – 10) Likert (1 – 10) Likert (1 – 10) Likert (1 – 10) Likert

Table 3 below presents the measurement items for each construct. The measurement items for attitude have included the benefit of the products’ dimension in shaping the individual's positive evaluation/perception toward participating in it. The measurement items for the subjective norm have involved the role of social pressure such as family, office mates, friends, and Takaful agents in influencing the behaviour. The third measurement item for perceived behavioural control includes an individual's perception of the ease or difficulty of participating in the products. Moreover, institutional trust is conceptualized to access individuals’ belief/trust toward the Takaful operators regarding regulation, legal recourse, etc. Finally, the intention construct examines an individual's probability of participating in Family Takaful (Solidarity) products.

The questionnaires in Table 3 were responded to by 272 academicians in seven Malaysian private Islamic Higher Education Institutions with 100 percent valid cases. Table 4 below summarizes the results of the descriptive analysis of respondents. The results consist of frequency and percentage for each question. Furthermore, Table 5 illustrates the descriptive analysis results for multi-scale items of each construct. The results include mean, standard deviation, skewness, and kurtosis values. Table 4 and Table 5 were extracted using SPSS version 26.

**Table 4:** Results for descriptive analysis of respondents.

Variable	Questions	Frequency	Percentage
**Q1**	**Gender**		
	Male	107	39.3
	Female	165	60.7

**Q2**	**Marital Status**		
	Married	47	17.3
	Single	225	82.7

**Q3**	**Age**		
	21 – 30 years	43	15.8
	31 – 40 years	122	44.9
	41 – 50 years	76	27.9
	> 50 years	31	11.4

**Q4**	**Highest education level**		
	Bachelor's degree	33	12.1
	Master's degree	153	56.3
	PhD	86	31.6

**Q5**	**Monthly income**		
	RM2501 – RM4000	97	35.7
	RM4001 – RM5500	60	22.1
	> RM5501	115	42.3

**Q6**	**Year of services**		
	Less than 5 years	112	41.2
	6 – 10 years	59	21.7
	11 – 15 years	51	18.8
	16 – 20 years	19	7.0
	> 21 years	31	11.4

**Q7**	**Name of Institutions**		
	Universiti Islam Selangor (UIS)	77	28.3
	Albukhary International University (AIU) International Centre for Education in Islamic Finance (INCEIF) Universiti Islam Antarabangsa Sultan Abdul Halim Mu`adzam Shah (UniSHAMS) Universiti Sultan Azlan Shah (USAS) Universiti Islam Pahang Sultan Ahmad Shah (UniPSAS) Universiti Melaka (UNIMEL)	11 6 47 59 31 41	4.0 2.2 17.3 21.7 11.4 15.1

**Table 5:** Results for descriptive analysis on multi-scale items.

Items	Mean	Std. Deviation	Skewness	Kurtosis
**Q8**				
ATT1	8.67	1.396	-0.877	-0.099
ATT2	7.86	1.703	-0.549	-0.641
ATT3	8.44	1.36	-0.518	-0.516
ATT4	8.01	1.51	-0.459	-0.635
ATT5	8.38	1.404	-0.688	-0.173
ATT6	8.46	1.495	-0.846	-0.152
ATT7	8.15	1.724	-0.746	-0.405
ATT8	8.68	1.373	-0.915	0.091

**Q9**				
SN1	6.52	2.59	-0.446	-0.702
SN2	7.08	2.28	-0.633	-0.263
SN3	6.72	2.468	-0.522	-0.451
SN4	6.25	2.275	-0.368	-0.32
SN5	6.51	2.273	-0.499	-0.281
SN6	7.64	2.167	-0.8	0.068
SN7	7.82	2.041	-0.909	0.412
SN8	7.21	2.189	-0.574	-0.333

**Q10**				
PBC1	7.4	1.924	-0.468	-0.506
PBC2	7.34	1.97	-0.415	-0.7
PBC3	7.74	1.899	-0.622	-0.293
PBC4	8.05	1.716	-0.675	-0.09
PBC5	6.85	2.261	-0.418	-0.592
PBC6	6.33	2.302	-0.131	-0.722
PBC7	7.15	2.197	-0.571	-0.416
PBC8	7.21	1.921	-0.454	-0.425

**Q11**				
IT1	8.1	1.502	-0.644	0.044
IT2	7.97	1.598	-0.52	-0.534
IT3	8.13	1.536	-0.556	-0.489
IT4	8.1	1.507	-0.47	-0.581
IT5	8.03	1.578	-0.543	-0.505
IT6	8.24	1.576	-0.793	0.095
IT7	8.01	1.589	-0.457	-0.474
IT8	7.93	1.564	-0.368	-0.691

**Q12**				
INT1	8.03	1.626	-0.629	-0.235
INT2	8.24	1.643	-0.856	0.22
INT3	8.26	1.606	-0.858	0.312
INT4	8.26	1.628	-0.805	0.104
INT5	8.19	1.641	-0.708	-0.295
INT6	8.21	1.696	-0.905	0.328
INT7	8.16	1.689	-0.715	-0.229
INT8	8.31	1.635	-0.7	-0.342

Table 5 below reports the indicators for descriptive analysis, including mean, standard deviation, skewness, and kurtosis. Mean, and standard deviation are used to measure the central tendency and dispersion, respectively. Based on the results, the mean and standard deviation values for all items are 68 percent within the suggested value, thus suggesting a normal central tendency. At the same time, skewness and kurtosis indicate the normality of the data. The suggested normality range value is +1.96 and -1.96 [15]. Thus, the data of the study fulfils the normality criteria.

The analysis proceeded with a measurement model for all multi-scale items (Q8 – Q12) using SmartPLS 3.3.7. The analyses included factor loading and average variance extracted for convergent validity. Moreover, internal consistency checking was also conducted using Cronbach's alpha and composite reliability. The study also continued with Heterotrait-Monotrait Correlation Ratio (HTMT) for discriminant validity, correlation analysis and the square root of AVE. Table 6 presents the said criteria of the measurement model. Some of the items (e.g., ATT6, ATT7, SN4, SN5 & PBC6) were deleted due to lower factor loading (<0.7). Table 7 indicates the results on HTMT (lower left triangle), correlation (upper right triangle) and the square root of AVE (in bold). All results fit the suggested criteria by most past literature.

**Table 6:** Results for measurement model for each construct.

Variable	Factor Loading	Cronbach's Alpha	Composite Reliability	Average Variance Extracted
**Q8** ATT1 ATT2 ATT3 ATT4 ATT5 ATT6 ATT7 ATT8	0.845 0.751 0.858 0.838 0.920 *Deleted* *Deleted* 0.886	0.923	0.940	0.725

**Q9** SN1 SN2 SN3 SN4 SN5 SN6 SN7 SN8	0.785 0.792 0.842 *Deleted* *Deleted* 0.815 0.805 0.711	0.881	0.910	0.628

**Q10** PBC1 PBC2 PBC3 PBC4 PBC5 PBC6 PBC7 PBC8	0.846 0.832 0.793 0.783 0.759 *Deleted* 0.781 0.709	0.897	0.919	0.620

**Q11** IT1 IT2 IT3 IT4 IT5 IT6 IT7 IT8	0.877 0.892 0.909 0.939 0.934 0.889 0.859 0.861	0.965	0.970	0.802

**Q12** INT1 INT2 INT3 INT4 INT5 INT6 INT7 INT8	0.893 0.907 0.938 0.947 0.942 0.957 0.954 0.847	0.975	0.979	0.854

**Table 7:** HTMT criterion, correlations, and the square root of AVE.

Constructs	ATT	SN	PBC	IT	INT
ATT	**0.851**	*0.432*	*0.624*	*0.672*	*0.663*
SN	0.473	**0.792**	*0.428*	*0.452*	*0.470*
PBC	0.681	0.474	**0.787**	*0.644*	*0.648*
IT	0.706	0.489	0.683	**0.896**	*0.659*
INT	0.696	0.503	0.687	0.674	**0.924**

Referring to the above table, the factor loading values for all measurement items are recorded between 0.709 and 0.957, which surpassed the suggested value of 0.70 [16]. Furthermore, Cronbach's alpha internal consistency values are recorded at 0.881 to 0.975. This value range indicates a good internal consistency between the constructs [16,17]. Lastly, the composite reliability and AVE exceeded the suggested values at 0.70 and 0.50, respectively.

Table 7 below illustrates the discriminant validity HTMT criterion results, Fornell Larcker (square root of AVE), and correlation analysis. The HTMT reports a value lower than 0.85 and thus has discriminant validity. The correlation between endogenous and exogenous variables for ATT, PBC and IT indicated a moderate positive correlation. Only SN indicates a low positive correlation. Finally, the Fornell Larcker indicators (square root of AVE) are higher than the correlation and thus validate the discriminant between the constructs.

Finally, the structural model was performed on all the constructs adopted from past literature works. This research methodology employs Structural Equation Modeling (SEM), which integrates two multivariate techniques: Confirmatory Factor Analysis (CFA) and Multiple Regression Analysis (MRA) [17]. SEM has garnered significant popularity in research circles for several reasons. It enables the statistical evaluation of complex processes and sophisticated concepts involving numerous exogenous and endogenous constructs [18].

**Fig. 1:**
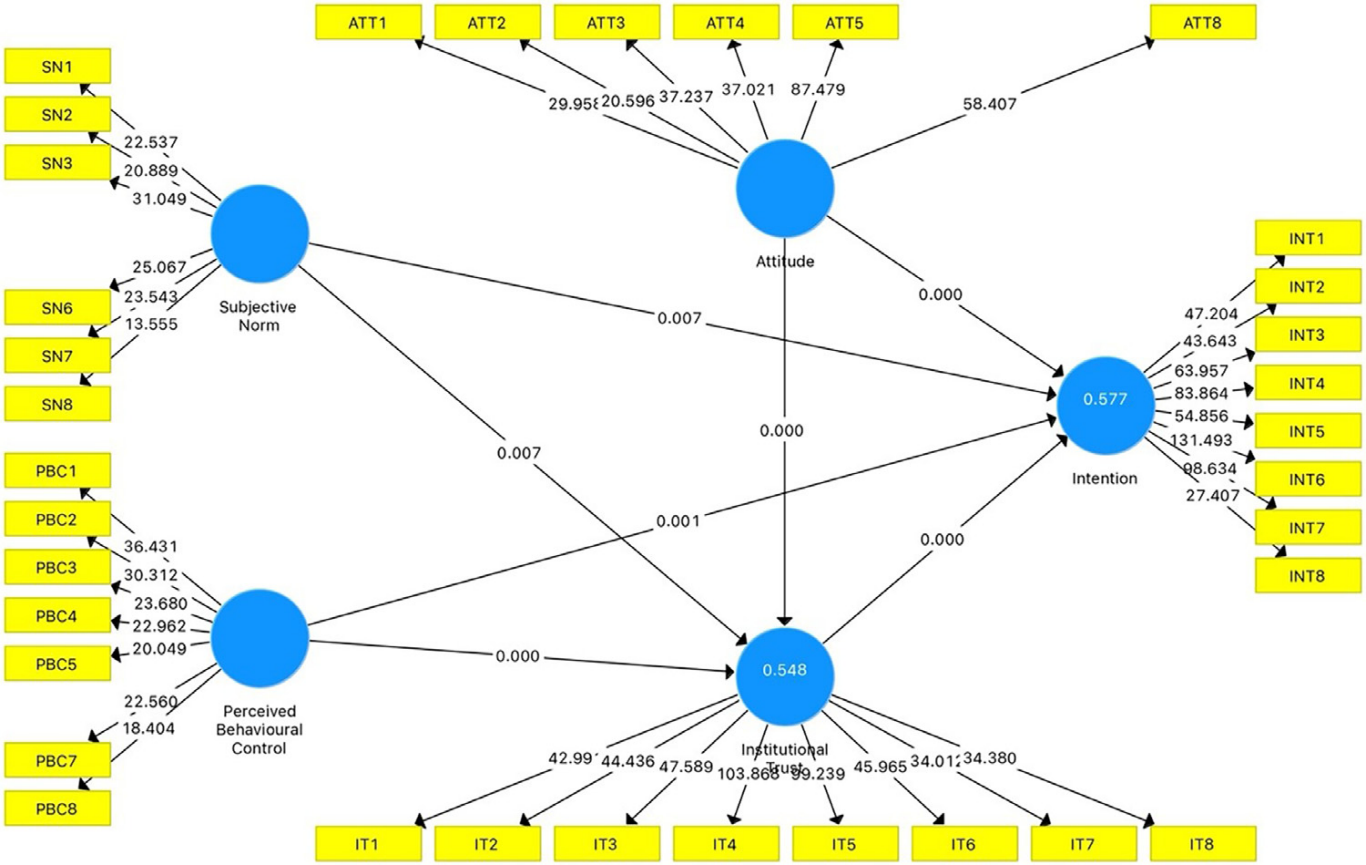
Structural model of the study.

As mentioned, the selected three constructs (attitude, subjective norm, and perceived behavioural control) are based on the Theory of Planned Behaviour. In contrast, the institutional trust construct is based on the Initial Trust Formation Model. Fig. 1 illustrates the result of the structural model of the study. The figure indicates that all constructs have a significant value of <0.05 toward the dependent variable.

The study further reports the model fit criteria using Standardized Root Mean Square Residual (SRMR) and bootstrap-based test (d_ULS and u_G). The SRMR criteria measure the standardized root mean square residual (SRMR) by transforming the sample covariance matrix and predicted covariance matrix into correlation matrices [19]. Meanwhile, the bootstrap-based test is used to perform exact overall model fit tests by statistically inferring the discrepancy between the empirical covariance matrix and the covariance matrix implied by the composite factor model. Table 8 below summarizes that all model fit criteria are accepted.

**Table 8:** Results for study's model fit.

Criteria	Suggested Value	Result	Interpretation
SRMR	<0.08	0.063	Accepted
d_ULS	<95%	0.878	Accepted
u_G	<95%	0.693	Accepted

## 4 Experimental Design, Materials and Methods

The first step of this study is begun by conducting an extensive review of the theoretical framework. This step determines the possible variables explaining the customer's intention to participate in Family Takaful (Solidarity) products. The review found the potential factors in shaping the customers’ intention, including attitude, subjective norms, perceived behavioural control and institutional trust. In the next step, a set of questionnaires was designed using a 10-point Likert scale, which has been empirically tested in past literature. All past empirical works fulfilled the reliability and validity of the constructs.

Step number three is to determine the sampling size. The academicians in seven private Islamic Higher Education Institutions in Malaysia were chosen. The sampling size determined as much as 265 academicians based on a population of 846 academicians in seven Malaysian private Islamic Higher Education Institutions and the sampling table from [20]. At a 95 percent confidence level, the dataset of 272 cases has a margin of error of ±4.89 percent in sample representativeness. The next step is to test the questionnaires developed on selected academicians to check their readability and comprehension. The fifth step is to proceed with the data collection on the unit of analysis. The questionnaires were distributed to the academicians using Google Forms and email invitations. Prior to the data collection, the authors received the academicians’ email from their respective institutions.

The data collection procedure received 272 respondents from the academicians in seven Malaysian private Islamic Higher Education Institutions. The collected data is continued with the data analysis. A comprehensive analysis was conducted, including a descriptive analysis of the respondents, descriptive analysis on the multi-scale items, reliability and validity using factor loading, Cronbach's alpha, composite reliability, an average of variance extracted, HTMT, correlation and square root of AVE. The analysis then proceeded with the structural model assessment as the goodness of fit was fulfilled, as suggested by the literature.

## 5 Limitations

The study's results provide compelling evidence in support of the proposed model. However, as with any empirical research, certain limitations warrant further investigation with a broader range of customers, including private-sector customers, to ensure the model's applicability can be appropriately generalized. It's worth noting that the sample used in the study may not fully represent the Malaysian population. Another limitation is that the study is a cross-sectional quantitative, meaning that data is only collected once. This constraint may be due to the study's time frame, and it's important to note that individuals’ intentions to participate in family takaful products can fluctuate over time due to personal circumstances, economic conditions, or market dynamics. As a result, this research may not account for temporal variations, potentially making the findings less relevant for understanding how intentions might evolve in the future. To address this limitation, a longitudinal study in the future with various customers’ demographic profiles may help accommodate changes in individual behaviour.

## Ethics Statement

All authors comply with the ethics procedure of the Universiti Utara Malaysia, Malaysia. All respondents were thoroughly informed about the content and the scope of the study before participation. Participation was completely voluntary, and participants cannot be identified. Also, this study does not require ethical approval from any organization.

## CRediT authorship contribution statement

**Mohd Faizuddin Muhammad Zuki:** Conceptualization, Methodology, Formal analysis, Investigation, Writing – original draft, Writing – review & editing, Visualization. **Nashirah Abu Bakar:** Supervision.

## Data Availability

Dataset on the Conceptualization of Institutional Trust on the Intention to Participate in Family Takaful Products (Original data) (Mendeley Data)
